# Prospects for arable farm uptake of Short Rotation Coppice willow and miscanthus in England

**DOI:** 10.1016/j.apenergy.2013.02.032

**Published:** 2013-07

**Authors:** Neryssa J. Glithero, Paul Wilson, Stephen J. Ramsden

**Affiliations:** Division of Agricultural and Environmental Sciences, School of Biosciences, University of Nottingham, Loughborough, Sutton Bonington Campus, LE12 5RD, United Kingdom

**Keywords:** Miscanthus, SRC, Farmer survey, Farm Business Survey, Bioenergy, Biomass supply

## Abstract

Biomass will play a role in the UK meeting EU targets on renewable energy use. Short Rotation Coppice (SRC) and miscanthus are potential biomass feedstocks; however, supply will rely on farmer willingness to grow these crops. Despite attractive crop establishment grants for dedicated energy crops (DECs) in the UK, uptake remains low. Drawing on results from an on-farm survey with 244 English arable farmers, 81.6% (87.7%) of farmers would not consider growing miscanthus (SRC), while respectively, 17.2% (11.9%) would consider growing and 1.2% (0.4%) were currently growing these crops. Farmer age, location, land ownership, farm type, farm size and farmer education level were not significant factors in determining acceptance of DECs. The main reasons cited for not growing DECs were impacts on land quality, lack of appropriate machinery, commitment of land for a long period of time, time to financial return and profitability. Reasons cited for willingness to grow DECs included land quality, ease of crop management, commitment of land for a long period of time, and profitability. Farmers cited a range of ‘moral’ (e.g. should not be using land for energy crops when there is a shortage of food), land quality, knowledge, profit and current farming practice comments as reasons for not growing DECs, while those willing to grow DECs cited interest in renewable energy, willingness to consider new crops, and low labour needs as rationale for their interest. Farm business objectives indicated that maximising profit and quality of life were most frequently cited as very important objectives. Previous research in the UK indicates that farmers in arable areas are unlikely to convert large areas of land to DECs, even where these farmers have an interest and willingness to grow them. Assuming that those farmers interested in growing DECs converted 9.29% (average percentage of arable land set-aside between 1996 and 2005) of their utilised agricultural area to these crops, 50,700 ha and 89,900 ha of SRC and miscanthus would, respectively, be grown on English arable farms. While farm business objectives were not identified as key determinants of DEC acceptance, enhanced information exchange through extension agents, providing market security and considering land reversion grants post-production are potential policy considerations.

## Highlights

► Survey of English arable farms to determine attitudes towards dedicated energy crops. ► 81.6% (87.7%) of surveyed farmers would not consider growing miscanthus (SRC). ► Farm/farmer characteristics were not significant factors in energy crop acceptance. ► Wide range of reasons given for farmers decisions related to energy crops. ► Farm business objectives not found to influence dedicated energy crop acceptance.

## 1 Introduction

To meet European Union targets on renewable energy use by 2020 (EU, Directive 2009/28/EU), bioenergy, including biomass, will play an important role [1]. A range of potential European bioenergy crops exists [2]; however, as feedstocks for second generation bioenergy, willow (in the form of ‘Short Rotation Coppice’, subsequently referred to as ‘SRC’) and miscanthus are the main biomass crops that are currently being considered by UK farmers. While forest residue also offers a source of bio-feedstock, it has been argued that crop biomass products will be required for a sustainable bioenergy industry to develop [3]. Moreover, Life Cycle Analysis indicates that these products offer the potential to reduce the environmental impact of energy production [4]. Miscanthus and SRC willow are dedicated energy crops (DECs), as opposed to crops with a possible dual purpose such as food and energy (e.g. wheat – grain and straw) and are perennial. In England, the energy crop scheme [5] was set up in 2007 to encourage farmers to grow SRC and miscanthus. The scheme provides grants that cover – in part – establishment costs; these are high in comparison to conventional arable crops [1]. The harvested biomass from both crops can either be used for power generation in the form of electricity (via combustion) or for biofuels (e.g. via lignocellulosic conversion to ethanol). However, establishment grants do not support the on-going costs of production; moreover, this type of subsidy may not target the optimal point in the bioenergy production chain [6]. For example, supporting location-specific bioenergy plants with government backed feedstock contracts to farmers, or an annual on-going subsidy to farmers may induce greater feedstock production than establishment grants alone. The focus of this paper is to gather information on the reasons behind farmers’ decisions in relation to the production of (or lack of interest in) SRC and miscanthus, including information on farmer objectives. We do this to gauge the potential supply of SRC and miscanthus in England from arable farms. This information is important if a sustainable energy system involving these crops is to be implemented in England and if policies are to be developed to best utilise or encourage the growth of these crops.

As perennial crops, miscanthus and SRC represent a departure from normal cropping patterns on UK arable farms which are predominantly based around annual crops grown in rotation to ensure good soil and crop health and performance. SRC willow can be first harvested four years after plantation, usually by stem cuttings, and can then generally be harvested every three years after this [7]. SRC has been identified as a feasible bioenergy system from an energy perspective which additionally offers environmental benefits over conventional energy production [8]. The amount of crop harvested varies with land quality, among other factors, and yields range between 21 and 27 oven dried tonnes per hectare (odt/ha) [9]. Estimates for the lifespan of SRC range between 22 and 30 years [7,10,11]. The costs for the establishment of SRC were estimated to be £1730 ha^−1^ [9] in 2012 of which 50% will be recoverable via the energy crops scheme grants. Every three years the farmer can expect a gross margin (value of sales less variable costs of production) of circa £720 ha^−1^ if the initial establishment costs are spread equally over the 21 year lifespan of the crop [9]. Miscanthus is usually propagated by planting sections of rhizome and can be first harvested towards the end of its second year after planting. Harvesting is then carried out annually and in the UK the crop is expected to have in the region of a 20 year lifespan [7,11] although estimates for this vary between 15 and 20 years [9]. Yields within the first years of establishment will generally be lower; after approximately five years yields of 12–16 odt/ha can be achieved [12,9] although this again is dependent on land quality. The establishment costs and gross margins noted below were taken from Nix (2011), a standard source for financial information in UK agriculture [9]. The establishment costs for miscanthus were estimated to be £2,462 ha^−1^ in 2012; again, 50% is recoverable via the energy crops scheme grant. Overall, the gross margin for this crop could vary between circa £324 ha^−1^ and £632 ha^−1^ a year, with establishment costs spread over a lifespan of 19 productive years. In contrast a winter wheat crop in England will generate a gross margin of between circa £395 ha^−1^ and £869 ha^−1^ per year, depending on feed or milling wheat grade outcomes and productivity on farm, although cereal crop prices are highly variable at present. The profitability of miscanthus in relation to more conventional combinable cropping that can be harvested with a combine harvester has been compared for farming in central France; the conclusion was that miscanthus was less profitable than combinable cropping, but could be highly competitive as a diversification enterprise on farm [13]. However, the cost of production calculated as cost per gigajoule of energy has shown that SRC crops and perennial grasses can have lower production costs than annual, straw-based crops [10].

In 2011, 3000 ha of SRC and 8000 ha of miscanthus were grown in England (relative standard error 10–20% and 5–10% respectively) [14]. For SRC this represents 0.03% of the utilised agricultural area (UAA) and 0.09% of the UAA for miscanthus. This contrasts with the 36% of the England UAA dedicated to cereal and oilseed crops [14]. It has been estimated that 17% of the South East of England and 39% of the East Midlands are potentially suitable for growing DECs and that overall 3.1 Mha of England is suitable for these crops [15]. A more conservative estimate of 362,865 ha (miscanthus only) is provided by [16] where various land and yield constraints are allowed for. Yield from this area of miscanthus is estimated to be 4.56 M odt which would potentially provide 6.5 M MW h of renewable electricity for the UK or 2.4% of total electricity demand in 2005 [16]. However, at present the uptake of these crops is lower than expected due to a variety of ‘barriers’ to adoption. These include: the uncertainty and extent of the financial return of these crops, particularly in relation to arable crop returns [17–19]; the reliance on a limited number of purchasers for the crops and the limited alternative market opportunities [17,20]; concerns relating to security of demand for crops that require a long term commitment [21,20]; and the lack of comprehensive information for these crops that is available to farmers or, more broadly, lack of farmer knowledge [17,22]. In an Irish study the differences between SRC and miscanthus have been evaluated and it was suggested that SRC willow is perceived to be more risky than miscanthus [23]. Whilst it has been noted that there are no absolute barriers to bioenergy in the EU, it is the non-technical challenges that are more likely to hold back production of suitable feedstocks [24]. A survey of 172 farmers in Ireland, from a wide range of farm types, showed that over 70% of farmers were interested in energy crops although the authors suggested that the method of dissemination for the survey had encouraged those interested in DECs to respond [22]. A motivation for adoption was the perceived profits for DECs. A study of the behaviour of German farmers found that farmers’ decisions were driven mainly by capital costs and the risk of investment, with non-financial objectives and sustainability issues being of limited influence [25]. The study also found that subsidies increased willingness to invest in bioenergy crops, as would be expected. The influence of farm and farmer characteristics in determining willingness to grow DECs has also been explored in a further Irish study [26], as well as American [27] and Swedish [28] contexts. Overall, no consistent linkages between farmer objectives, behaviours, characteristics or farm physical features are evident from the literature. However, a priori, it would be expected that the attitudes and objectives of farmers would play some role in determining their attitudes towards growing DECs.

Given the potential importance of DECs in contributing towards meeting renewable energy targets, this paper examines the objectives of, and rationale behind, farmers’ decisions on arable farms relating to SRC and miscanthus and the potential supply of these crops in England from these farms. Specifically, the aim of the paper is to (a) describe the survey methodology adopted; (b) indicate the numbers of farmers willing to grow SRC and miscanthus and analyse these responses in relation to a number of farmer characteristics; (c) identify farmer attitudes, objectives and the main reasons given for growing and not growing these DECs; (d) estimate potential areas of these crops that could be grown on arable farms in England based on the survey results and (e) draw conclusions from these results in relation to the potential barriers/incentives identified to growing and not growing DECs on arable farms and potential bioenergy supply. The survey design and methodology is outlined in Section 2. The key results from the survey in relation to DECs and on farm objectives are shown in Section 3 with Section 4 containing a discussion of these in the context of the UK bioenergy sector. Section 5 draws together and summarises the main conclusions of the study. The approach complements the estimated projections made for cereal straw in Glithero et al. [29].

## 2 Methods

To understand the potential that SRC and miscanthus have as biomass energy sources it is necessary to identify the willingness of farmers to grow these DECs and identify the motivating factors behind these decisions. Glithero et al. [30] give a more detailed overview of the methodology employed; in brief a survey was undertaken in England on arable farm types (Cereals, General Cropping, Mixed) which gathered information on straw use, straw volumes baled, crop cultivations, cereal variety choice, straw incorporation, contract implications of bioethanol feedstock production and dedicated bioenergy crops. The final survey was carried out in conjunction with the Farm Business Survey (FBS) by Rural Business Research ROs (Research Officers) in on-farm interviews between February and November 2011. The farms surveyed were a sub-sample of the FBS sample (approximately 46% of the farms within the arable farm types mentioned above) distributed across the eight Government Office Regions (GORs) of England and three size groups within the farm types stratified by sample numbers within the FBS. Farms within the FBS are representative of the national population of farms in England based upon the returns to the Defra (Department for the Environment, Food and Rural Affairs) annual June Survey. The questions of particular relevance for this paper relate to the farmer’s willingness to grow SRC and miscanthus, the importance of different factors in their decision on growing DECs and on their objectives for their farms (see Appendix A). Overall there were 244 completed farm returns for these questions. The number of farms surveyed in each farm type and GOR is given in Table 1.

The data collected on willingness to grow SRC and miscanthus has been analysed with respect to categories for farmer age, location, land ownership, farm type, farm size and farmer educational attainment using data from the FBS, with Chi-squared tests undertaken to test the hypothesis that these factors do not play a role in determining farmer attitudes with respect to these crops. The farmer age groupings for miscanthus were: under 44, 45–54, 55–64 and over 65 years and for SRC were under 54, 55–64 and over 65 years (slight differences were needed in the grouping for the two crops due to Chi-squared test requirements). The farmer’s education was classed as: school level only (GCSE’s, A-levels, Apprenticeships and other), college level or university level (degree or postgraduate). Land ownership was determined by the percentage of the land owned by the farmer which had two groupings: below 50% and 50% or above ownership. The farm types and sizes were taken from the FBS and were Cereals, Mixed, General Cropping, Large, Medium and Small and the farm location was based on the EU region of the farm: North England, West England and East England. Under the assumption that arable farmers would not convert all of their UAA to DECs, but would grow these crops on their least productive land [17,31], a realistic land conversion ratio based upon typical ‘set-aside’ rates has been used. During the 10 year period 1996–2005, set aside in the UK constituted 9.29% of arable area (Nix, various [32]). On the assumption that 9.29% of the UAA on a farm is converted to these crops, where a farmer was willing to grow, or is already growing these crops, the potential area of these crops that could be grown was calculated using the data aggregation method outlined in Glithero et al. [30]. This gives a potentially achievable national supply of these crops for bioenergy purposes from English arable farms.

Farmers were asked to select from a range of factors those that were important in their decision making regarding DECs. Due to the small number of responses from farmers already growing these crops the results for these farmers were combined with those that were willing to grow DECs. Hence there were two groups for the analysis of the important factors in the decision making process. In addition to the factors provided, farmers were able to list additional factors involved in their decisions if they wished to do so.

To investigate the potential objectives that farmers may have on arable farm types in England, farmers were asked to rank the importance of four objectives that were provided, from “Very Important” to “Very Unimportant”. These were chosen to give a range of farm objectives related to financial, environmental, and family and ‘life’ aspects and in general were worded to allow for a broad interpretation by farmers. The objectives given were: maximising profit, environmental and land stewardship, stewardship for the next generation and quality of life. These represent a succinct survey data capture technique to assess the importance of the major farmer objectives as previously identified by key comments from English farmers in qualitative interviews [33]. Farmers were able to add additional farm objectives if they wished to.

## 3 Results

### 3.1 Willingness to grow SRC and miscanthus

Farmers were asked if they would be willing to grow SRC and miscanthus and could respond with ‘yes’, ‘no’ or ‘already growing these crops’. In the background notes given to the Research Officers who carried out the on-farm interviews it was indicated that questions related to SRC were specifically related to SRC willow. Of the 244 responses to this question for miscanthus, 81.6% responded that they would not be willing to grow this crop, 17.2% responded that they would be willing and 1.2% noted that they already grew the crop. For SRC, 87.7% responded that they would not be willing, 11.9% responded that they would be willing and 0.4% noted that they already grew this crop. These results concur with evidence from Ireland where miscanthus was also identified to be of greater interest to farmers than SRC [22]. Analysing the data for the two crops together, 10.7% of farmers would be willing to grow both crops and 79.9% of farmers would not be willing to grow either crop. These data were then combined with that for farmers’ age and education, farm ownership information, farm type and size and farm location from the FBS and chi-squared tests performed. There was no significant effect of farmers’ age, education level, farm ownership, farm location (EU region), farm type and size on willingness to grow either SRC or miscanthus: *p*-values given in Table 2.

### 3.2 Factors influencing SRC and miscanthus decision making

**Fig. 1:**
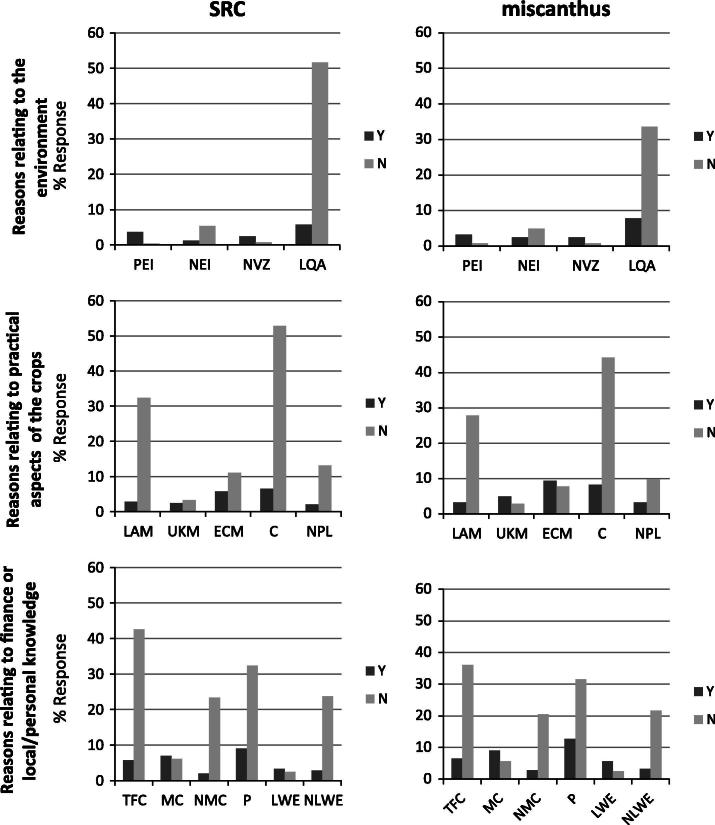
Percentage responses from those that would and would not be willing to grow SRC and miscanthus. PEI positive environmental impact, NEI negative environmental impact, NVZ nitrate vulnerable zone restrictions, LQA land quality aspects, LAM lack of appropriate machinery, UKM use of known machinery, ECM ease of crop management, C committing the land for a long time period, NPL needing permission from landlord, TFC time to financial return on crop, MC market for crop, NMC no market for the crop, P profitability, LWE local working example and NLWE no local working example.

The reasons given for being willing or not to grow miscanthus and SRC are shown in Fig. 1. The response profile of SRC is similar to that of miscanthus. Of the environmental reasons, land quality aspects (e.g. damage to drains, cost of land change back to an agricultural use) was the most frequently cited environmental reason for not growing SRC and miscanthus. This also featured highly in the reasons for growing these crops along with the positive environmental impact factor. In terms of the practical reasons selected, the main ‘no’ reasons were the lack of appropriate machinery and the committing of land for a long time period. The key ‘yes’ reasons were committing land for a long time period and the ease of crop management. The time to financial return and profitability were the main financial, market and knowledge reasons against growing these crops. Conversely, profitability was also the main reason for growing these crops, although the second highest financial, market and knowledge reason cited for growing SRC and miscanthus was ‘a market for the crop’. Respondents were able to add additional reasons that were important in their decision making and 52 farmers provided information. The additional reasons given by farmers willing to grow (or already growing) SRC and miscanthus can be seen in Table 3. The majority of the comments can be categorised as general interest or ‘moral concern’ comments, or can be grouped as relating to land and resource management on-farm. Additional reasons from farmers not willing to grow these crops were also grouped into categories. The most common responses for not growing SRC and miscanthus were related to the desire to maintain current farming activities, Table 4.

### 3.3 Potential supply

**Fig. 2:**
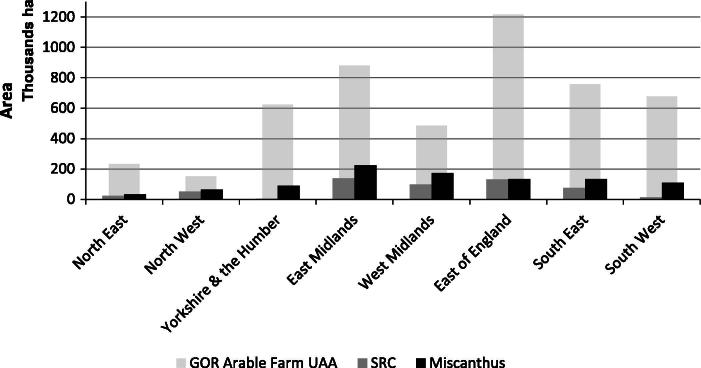
Potential area of SRC and miscanthus grown assuming 100% of UAA, for these farm types, is converted into these crops.

To calculate a realistic potential supply of SRC and miscanthus from arable farm types in England it was assumed that 9.29% of the UAA (the Utilisable Agricultural Area) could be converted to these crops on farms where respondents were willing to grow or were already growing these crops. From this the area that could potentially be converted in England was calculated, using the aggregation method mentioned earlier. Under these assumptions, the largest area of these crops would be grown in the East Midlands; however it is in the North West that DECs could constitute the largest percentage of the GOR arable farm type area. Overall, the potential area of SRC and miscanthus that could be grown on arable farm types is 50,700 ha and 89,900 ha respectively. However, if 100% of the UAA was converted to DECs where farmers were willing to grow, or were already growing these crops, considerably larger areas of DECs could be produced. Fig. 2 shows the theoretical maximum areas of SRC and miscanthus under this assumption, demonstrating that 545,700 ha of SRC and 967,500 ha of miscanthus would represent an absolute upper bound of production from arable farms in England, even where 100% UAA conversion was undertaken.

### 3.4 Farm business objectives

**Fig. 3:**
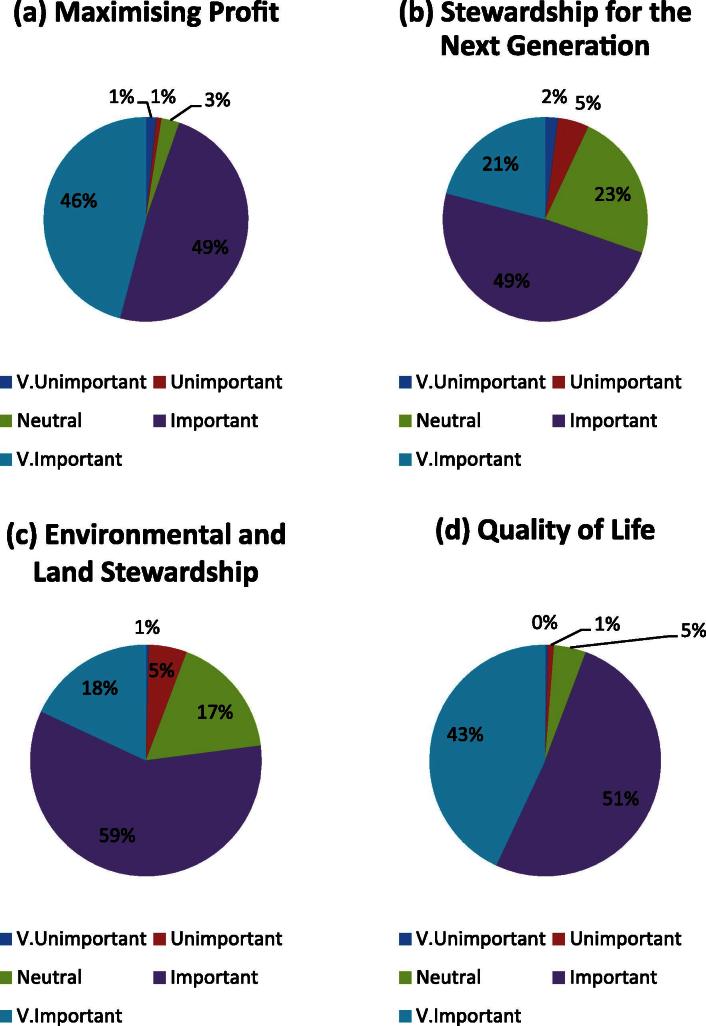
The importance profiles of the four on farm objectives; maximising profit, stewardship for the next generation, environmental and land stewardship and quality of life.

Farmers were asked to rank the importance of four given objectives (objectives listed in the methods section) from ‘Very Important’ to ‘Very Unimportant’; in addition supplementary objectives, where they wished to provide further detail, were recorded. The responses from the farmers for the given objectives can be seen in Fig. 3, which shows the percentage of responses for each of the importance rankings for each of the objectives. Fig. 3a and d respectively indicate the ‘Very Important’ nature of maximising profit and quality of life, cited by 46% and 43% of farmers respectively, with 49% and 51% respectively additionally selecting these factors as ‘Important’. Stewardship for the next generation and environmental and land stewardship were cited as ‘Important’ or ‘Very Important’ by 70% and 77% of farmers respectively. Analysing the responses gathered, most farmers recorded a ‘Very Important’ or ‘Important’ answer for the four objectives given (overall 85% of responses from farmers where in these categories).

**Fig. 4:**
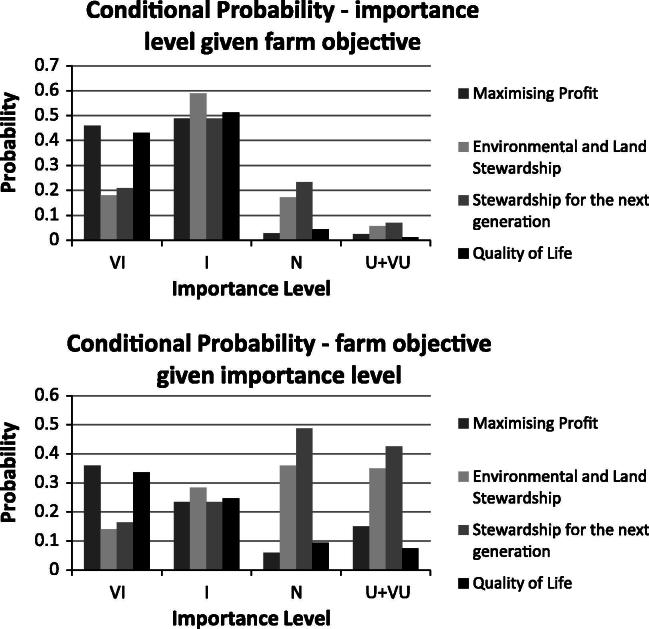
Conditional probabilities: VI – very important, I – important, N – neutral and U + VU – unimportant and very unimportant combined.

The percentage of responses given for each of the five importance options given to farmers was calculated for each of the four farmer objectives. This ‘profile’ is significantly different (*p* < 0.001) between the four farmer objectives given (maximising profit, stewardship for the next generation, environmental and land stewardship and quality of life). Fig. 4 shows ‘conditional’ probabilities: the probability of a certain importance level occurring if the farm business objective is known or the probability of a certain farm objective occurring if the importance level is known. For example, the probability of a ‘Very Important’ response given that farm business objective was maximising profit. Overall the conditional probabilities show that maximising profit and quality of life were more likely to be scored as ‘Very Important’ in comparison to the other two objectives given. The probabilities of an objective being scored as ‘Important’ is approximately *P*(Important|objective) = 0.5 for all the objectives given.

Given the reasons cited for willingness and lack of willingness to grow DECs, the responses for farm business objectives were analysed to test the null hypothesis that farm business objectives do not influence attitudes towards growing DECs. To undertake this analysis, the ‘Very Important’ responses were compared with the ‘willingness to grow SRC and miscanthus’ responses; no significant difference was found. Previous research has indicated a link between environmental objectives and willingness to grow DECs [19,22]. These data were therefore additionally analysed combining the ‘Very Important’ and ‘Important’ objective responses for environmental and land stewardship to test the null hypothesis of no difference between willingness to grow SRC and or miscanthus, and the farmer ranking of environmental objectives. For both SRC and miscanthus, no significant difference was observed (*p* = 0.974 SRC; *p* = 0.979 miscanthus). This result is potentially a function of the majority of objective responses being ranked ‘Very important’ or ‘Important’ leading to modest number of objective responses in the remaining rank categories.

## 4 Discussion

The majority of farmers surveyed were not willing to grow SRC or miscanthus, with land quality, the commitment of land for long time periods and the time to financial return being the main reasons cited for this decision. Adams et al. [19] report similar findings, with profitability of DECs in relation to current investments, uncertainty of financial return and land availability being important barriers to DEC production. Of the farmers willing to grow either/or both of these DECs, positive environmental impact, commitment of land for a long time period and profitability of the crops were the main reasons given. Adams et al. [19] note that negative environmental impacts of feedstock production is also a barrier to DECs, while ‘other environmental benefits beyond CO_2_ reductions’ represent a driver for DEC feedstock production, hence the environmental impacts of DECs are perceived as both barriers and drivers [19] highlighting the diversity of farmer attitudes towards DEC production. Augustenborg et al. [22] found that on average, potential energy crop adopters ranking of bioenergy production as being ‘beneficial to the environment’ was significantly greater than the average rank provided by non-adopters. Our findings indicate that for arable farmers in England, there is no link between environmental objectives and willingness to grow DECs, in part, contrasting with these previous finding [19,22]. Additional reasons against growing DECs related to lack of suitability to current farming activities; this was also noted as a barrier to bioenergy implementation by Roos et al.’s [34] analysis of critical factors relating to bioenergy implementation in five world bioenergy case studies. The profitability of DECs relative to current arable crops was cited as a key reason for not growing energy crops, as also found by Adams et al. [19] in their online survey. Our results demonstrate no links between various farm and farmer characteristics and willingness to grow DECs. Paulrud and Laitila [35] also found that the ownership of land, type of farming and the amount of set aside land (land retired as part of the operation of the Common Agricultural Policy at the time) had an insignificant effect on farmers’ willingness to grow energy crops, while farm size, farmer age and farm location influenced the willingness to grow energy crops [35].

Based on our assumptions (farmers’ willingness to grow and assuming 9.29% of the UAA on representative farms were converted) there would be potential for 50,700 ha of SRC and 89,900 ha of miscanthus on arable farm types in England. These estimates represent a large increase on the respective current production levels of circa 3000 and 8000 ha [14]. However, the estimates presented above are much lower than the 3.1 Mha available for DECs suggested by Haughton et al. [15]. Lovett et al. [16] calculated that 3.12 Mha of England was suitable for growing miscanthus although, as noted, when additional constraints related to prime agricultural land, yield considerations and the Biomass Strategy target were applied, this was reduced to 362,859 ha. Under the theoretical assumption that arable farmers interested in or currently growing DECs converted 100% of the UAA to these crops, our estimates remain substantially lower than the headline estimates in the literature [15,16]. Previous studies have suggested that small scale planting within a farm’s UAA will be more likely in certain regions of England, particularly given SRC’s modest relative economic returns [31]. There are also regional variations in the potential crop areas from our results. Moreover, the proportional area of arable farm types that could be converted to DECs differs, with the North West and the West Midlands representing areas of greater potential. These regions are associated with relatively high levels of precipitation and associated grass-based livestock production systems. The western areas of England and Wales are also regions where higher yields for SRC and miscanthus are generally achieved [36].

The results presented indicated no significant difference between farmer objectives and attitudes towards growing DECs. However, in some cases, these personal factors are likely to drive the decision as to whether or not to grow these crops, as has been previously argued by Karp et al. [31]. Breen et al. [26] identified that in an Irish context, agricultural education level, farm size and farm system significantly influenced the likelihood that farmers would grow energy crops; however, a range of other farm and farmer characteristics, including general educational attainment, farm profit, farm tenure status, farmer age, solvency of the farm business and contact with extension agents were insignificant determinants of willingness to adopt. Valendia et al.’s [27] assessment of switchgrass producers in East Tennessee found that following the expiry of supply contracts, farmers’ willingness to make long term commitments to growing the crop were likely to be influenced by community perceptions about bioenergy crops and support from extension workers. Within a Swedish context, Jonsson et al. [28] note that ‘values’, legal conditions, economic factors and knowledge are key determinants of farmer decisions about whether or not to grow DECs. Hence, while economic and land quality aspects arguably drive DEC decision making, our study has not demonstrated farm or farmer characteristics associated with DEC acceptance. Moreover, evidence from both the UK and international studies does not indicate clear consistent patterns between DEC choice or rejection and farmer characteristics. With respect to policy initiatives, such lack of clear linkages between farmer characteristics and DEC uptake are likely to hinder efficient policy delivery mechanisms to enhance the area of DEC produced on English arable farms.

The analysis presented herein indicates that uptake of DECs amongst English arable farmers is possible, with our estimates relying upon the relatively modest proportion of arable farmers that are willing to consider growing DECs converting typical set-aside rates of land to DEC production. The potential estimates based upon 9.29% UAA conversion to DECs are arguably representative of potential land conversion because, in practice, interested farmers would be more likely to convert small areas of their farms to DECs, particularly in the more arable-based areas of England [31]. Evidence from previous research indicates that availability of information from extension officers [27] plays a role in determining uptake of DEC, alongside community perception of growing the crops and farmer values [28]. Hence, while establishment subsidies for DECs will play a role in encouraging farmers to grow these crops, additional factors such as enhanced information delivery and exchange via an extension network will influence decision making together with the influence of farming community values and views towards the replacement of typical arable crops by DECs.

One major advantage of the current study in relation to previous research based upon survey or focus group approaches [17,19] in relation to DEC’s in England is that these data have been directly linked with the Farm Business Survey (FBS) data; a national annual survey into farm businesses that collects data on cropping areas, financial inputs and outputs, farmer characteristics, farm locations and some environmental factors. The FBS is commissioned by Defra and is used to assess the economic state of the agricultural industry within England. Although the survey outlined in this paper was carried out on a (substantial) subset of the farms included in the FBS, the linkages between the two surveys have permitted data aggregation to a national level in a manner consistent with current government methods, providing additional information than could not be drawn from stand-alone survey data. Moreover, embedding the survey within the FBS ensured that the farmer responses to the survey include those with both an interest in DECs and those with little or no interest in DECs. This approach arguably contrasts with previous studies examining attitudes towards DECs [19,17,22] whereby issues of sample bias potentially exist, with farmers who have a greater interest in the subject matter of the survey, or methodology of data capture (Adams et al. [19], web-based; Sherrington et al. [17], focus group based), being more inclined to respond to, or take part in, the research [37]. However, our findings broadly concur with these previous studies, reinforcing both the complexity and range of issues which are likely to affect bioenergy feedstock supply from UK farmers. The results presented herein add considerable validity to previous findings and can therefore be used to help inform governmental policies relating to forecasting the growth or development of SRC and miscanthus feedstock supplies and sustainable bioenergy systems more generally.

The implementation of bioenergy systems crucially relies upon a sustainable feedstock supply base [22]. Our findings demonstrate that most arable producers in the England would not consider growing DECs, with a range of financial, practical and environmental factors central to farmer attitudes towards the production of crops such as miscanthus and SRC. Policy makers seeking to implement bioenergy systems based upon DEC supply therefore need to consider policies to overcome these issues. For example, support towards resurrecting land quality post DEC production through ‘land reversion’ grants, reducing the cost of machinery required to produce DECs and reducing the financial risks of producing DECs over the longer term [22] (perhaps through government-underwritten industry contracts) represent potential policies to encourage DEC production. A further option would be improved provision of advisory support [27]. However, as shown by the responses of many of the FBS farmers, increased production of DECs would divert resources away from other land uses.

## 5 Conclusion

While biomass is likely to play a role in the UK meeting the EU renewable energy targets, the production of DECs will crucially rely upon farmer acceptance of these crops. With current uptake remaining low even in the presence of substantial crop establishment grants, eliciting farmer attitudes towards these crops is necessary if policy makers are to further encourage DECs. On the basis of a large scale on-farm survey of English arable farmers, contemporary attitudes towards DEC acceptance suggest that the majority of these farmers would not consider growing DECs, with a range of financial, land quality, practical and moral considerations lying behind their cited preferences. A small percentage of English arable farmers indicated a willingness to consider growing these crops (17% for miscanthus, 12% for SRC), although only large scale conversion of land from current use to DEC production would lead to substantial areas of DEC production, and current evidence indicates that this is unlikely to occur. In order to meet the EU renewable fuel target, policy makers seeking to enhance DEC production need to be both aware of the limited potential for DEC production on English arable farms and invest in extension activities [27] to engage in knowledge exchange with farmers along with provision of the financial and market security [22] required to incentivise farmers towards these crops. Without these types of initiative, the barriers to DEC production remain substantial.

## Figures and Tables

**Table 1:** Number of survey respondents by farm type and government office region.

GOR	Cereals	General cropping	Mixed
North East	8	1	7
North West	7	5	4
Yorkshire and the Humber	12	5	10
East Midlands	30	9	7
West Midlands	5	8	7
East of England	29	24	9
South East	20	3	10
South West	9	2	13

**Table 2:** *P*-values from the Chi-squared tests.

	SRC	Miscanthus
Farmer age	0.30	0.15
Location		
EU region	0.63	0.23
Land ownership	0.97	0.69
Farm		
Type	0.56	0.93
Size	0.91	0.68
Education level	0.76	0.79

**Table 3:** Additional comments for growing SRC and miscanthus. SRC – yes (6 comments), miscanthus – yes (5 comments) – Already Growing (AG) (1 comment).

Category	Segment	SRC	Misc	Quotes
Yes	Interest and “Moral”	3	2	*“Moral stand point for growing energy crops”*
				*“Never given it any thought, but would be interested to have a look”*
				*“Interested in renewable energy sources”*^a^

	Land and resource management	2	2	*“Long term weed control in crop e.g. blackgrass”*
				*“Low labour input required”*

	Other	1	1	*“Not very keen though”*

AG				*“Energy payment received and within the set-aside area”*

a Only applicable to SRC.

**Table 4:** Additional comments for not growing SRC and miscanthus. SRC – No (43 comments), miscanthus – No (45 comments).

Segment	SRC	Misc	Typical comments – summarised	Selection of quotes
Interest and “Moral”	6	6	• Not interested	*“No interest in growing these crops”*
			• Moral point against using land for energy crops	*“Should not be using land for energy crops when there is a shortage of food in the world”*

Current farming activities	17	17	• Does not fit with organic systems	*“Need all land for grain for fat cattle and sheep”*
			• Happy with/committed to current cropping	*“No synergy with current farming activities”*
			• Does not fit with current activities	
			• Need straw for livestock/bedding	
			• Already growing miscanthus/SRC	

Land and soil	8	8	• Not enough land	*“Only got a small acreage, good land better for growing food crops*”
			• Soil/land not suitable	*“Not enough land”*
			• Whole farm needed to be converted	
			• Good land for agricultural crops	

Knowledge	6	7	• Looked at but decided against	*“Previously investigated and decided against”*
			• Lack of knowledge of this crop	*“Lack of personal knowledge of the crops”*
			• Personal observations	

Profit	2	3	• Profitability relative to other enterprises	*“Price of wheat and OSR are good“*
			• Good arable crop prices	*“Profitability relative to other crops”*
			• High cost of rhizomes^a^	

Other	4	4		*“Age”*
				*“I am a farmer, not a woodman”*^b^
				*“SRC needs to dry after cutting and before being sent to the power plant. Farmer has no room or facilities to dry the crop”*^b^
				*“Poor track record of purchasing companies”*
				*“Become Invasive”*^a^
				*“Proximity to urban area (potential fire risk)”*^a^

a Only applicable to miscanthus.

b Only applicable to SRC.
